# Incidence and clinical outcomes of extended duration support in patients with Impella 5.5 - Analysis from the LOQI Registry

**DOI:** 10.1016/j.jhlto.2026.100586

**Published:** 2026-05-06

**Authors:** David Kaczorowski, Vidang P. Nguyen, Aasim Afzal, Scott C. Silvestry, Ahmad Zeeshan, Naveena Yanamala, Ray Matthews, Rothy Chhim, Sanjeev Aggarwal, Sami I. Somo, Gavin W. Hickey

**Affiliations:** a University of Pittsburgh Medical Center, Venetia, PA; b Providence, Portland, OR; c Baylor Scott & White Health, Plano, TX; d University of Arizona, Tucson, AZ; e AdventHealth, Orlando, FL; f Rutgers Health, Robert Wood Johnson Medical School, New Brunswick, NJ; g Keck School of Medicine of USC, Los Angeles, CA; h Johnson & Johnson MedTech, Danvers, MA; i University of Pittsburgh Medical Center, Pittsburgh, PA

**Keywords:** Cardiogenic Shock, Impella 5.5, Mechanical Circulatory Support, Extended support, Long duration support, Axial flow pump, Bridge to recovery

## Abstract

**Purpose:**

Impella 5.5 pumps are increasingly used in cardiogenic shock (CS) for extended hemodynamic support (>14 days). Rates of adverse events have not been well-characterized during longer durations of support. Therefore, we sought to define patient characteristics and adverse event rates in short versus extended duration of Impella 5.5 support (DOS).

**Methods:**

Baseline demographics, clinical characteristics, and serious adverse events defined as death or serious deterioration of health (Serious Adverse Event (SAE): hemolysis, stroke, renal failure, and vascular complications) were analyzed. SAE exposure adjusted event rates were compared between patients supported ≤14 days (short DOS cohort) and >14 days (extended DOS cohort).

**Results:**

Among the 2262 patients enrolled across 31 sites in the LOQI (Long-Term Outcome and Quality Impella) registry, 443 patients were supported with the Impella 5.5. Compared to the extended duration cohort, the short duration cohort were older, had a greater proportion of women, and had a higher incidence of hypertension. Event adjusted exposure rate (EAER) was significantly lower in the extended versus short DOS cohort for cumulative SAEs, (EAER: 0.0776 vs. 0.0235 events/day; p<0.001) as well as each of the individual components: hemolysis (0.011 vs. 0.0029), stroke (0.0086 vs. 0.0024), renal failure (0.0177 vs. 0.005), vascular complications (0.0124 vs. 0.0053), and bleeding (0.0235 vs. 0.0061).

**Conclusions:**

In a real-world setting, extended use of Impella 5.5 for >14 days did not increase the rate of serious adverse events and was able to provide hemodynamic support to a heterogenous group of patients presenting with cardiogenic shock with stable device performance.

## Introduction

Impella 5.5 pumps are increasingly used to support patients in cardiogenic shock for extended durations of support (>14 days). In an analysis of the United Network for Organ Sharing (UNOS) database,^1^ the median duration of support with Impella 5.5 was 19 days for 212 patients who used it as a direct bridge to isolated heart transplant. The UNOS registry analysis concluded that extended duration of use of Impella 5.5 as a bridge to transplantation did not adversely impact post-transplant outcomes. The Cardiogenic Shock Working Group (CSWG)^2^ found that the median duration of support with Impella 5.5/5.0 was 12.9 days (IQR: 6.8 – 22.9) in 754 cardiogenic shock patients. The CSWG stated that the use of the high-capacity Impella device (Impella 5.5/5.0) can provide sufficient hemodynamic support for longer durations, allowing for mobility, rehabilitation, and informed patient discussion about different treatment options.^2^ Another analysis from CSWG concluded that patients supported for longer than 14 days had favorable outcomes, especially as a bridge to heart transplant.^3^

A longer duration of use with Impella 5.5 is important to allow some cardiogenic shock patients to achieve hemodynamic stability, restore end-organ function, and for the care team to consider the best next stage of treatment. The use of temporary mechanical circulatory support (tMCS) in cardiogenic shock aims to stabilize hemodynamics, restore end-organ function, and metabolic parameters, with the goal of promoting myocardial recovery and gradually weaning the patient off tMCS to achieve native heart survival.^4^ Extended duration support with Impella 5.5 allows for stabilization, rehabilitation, and end-organ recovery in patients who subsequently require transition to durable replacement therapies with transplantation or durable left ventricular assist device (LVAD placement).^5^, ^6^ Recent research by Pieri et al. highlighted the role of minimally invasive tMCS devices, such as Impella 5.0/5.5, in managing new-onset acute heart failure.^7^ These devices offer rapid improvement in perfusion and unloading of the ventricle, providing clinicians with the necessary time to decide on the most appropriate treatment path. While the benefits of the Impella 5.5 in supporting patients acutely, complications are prevalent in this patient population and associated with worst outcomes. Adverse event associated with the Impella 5.5 include stroke, hemolysis, vascular complication, and kidney damage.^1^, ^3^, ^8^

With the increasing use of Impella 5.5 for extended duration support, we sought to analyze patient characteristics in an all-comer population and characterize adverse event rates as compared to short duration support. To assess the risk benefit profile of Impella 5.5 used for a duration longer than 14 days, clinical outcomes were analyzed using the Long-Term Outcome and Quality Indicator (LOQI) Impella Registry.

## Methods

### Study design

The Long-Term Outcome and Quality Indicator (LOQI) Impella Registry is a prospective, multi-center, observational, records review study of patients supported with all US commercially approved Impella devices including CP, 5.0/5.5, and RP devices (Heart Recovery, Johnson and Johnson MedTech, Danvers, MA). The purpose of the ongoing LOQI Registry is to capture observational data from consecutive patients to allow for real-world surveillance of key safety events, identification of best practices, support ongoing regulatory submissions, and to stimulate hypothesis-generating research. The study is conducted under an IRB-approved Waiver of Informed Consent and Health Insurance Portability and Accountability Act (HIPAA) Waiver of Authorization.

Subjects were enrolled in the registry once they underwent insertion of an Impella at the study sites. Patient demographics, medical history, medications, baseline hemodynamics, laboratory and echocardiographic data were collected from the electronic medical records. Adverse events were verified by site investigators, which included hemolysis, renal failure, stroke, vascular complication, and BARC ≥3 bleeding (Supplemental Table 1). Adverse events that were reported as both bleeding and vascular related were combined as a single event. Survival was assessed at hospital discharge and thereafter through 1-year post discharge.

### Analysis population

Between March 2022 and December 2024, 2262 subjects were enrolled across 31 sites in the LOQI Registry. The majority of sites were VAD and/or Transplant Centers. Of the 2262 patients, 443 were supported with the Impella 5.5. Patients included in the analysis were consecutively enrolled.

### Statistical analysis

Baseline characteristics were summarized using standard summary statistics, including frequencies, percentages, means and medians. Measures of variation were presented as mean ± standard deviation and/or median [25th, 75th]. Categorical variables were compared using Fisher’s exact or Chi-square test as appropriate. Continuous variables were compared using t-test or Wilcoxon rank sum test as appropriate.

Serious adverse events (SAE) were reported as counts and proportions. Exposure-adjusted event rates (EAER) from each duration of support stratification were evaluated as the total number of SAE reported divided by the total days of Impella 5.5 support in each stratification. Comparison of EAER was performed using a Poisson regression model with the number of events as the dependent variable and log (subject total duration of Impella 5.5 support) as the offset. The EAER 95% confidence interval (CI) was evaluated using exact Poisson confidence limits.

Native Heart Survival (NHS) was defined as survival to discharge without heart transplantation or durable LVAD during the index hospitalization. Survival was reported at explant, discharge, 30 days and 90 days post explant. A forward imputation method was used for patients with deaths prior to their 90-day follow-up for each subsequent visit. Kaplan-Meier survival curves for the occurrence of all-cause mortality from time of implant and conditional survival at discharge were plotted. Hazard ratio and 95% confidence intervals are reported, with a log-rank test used to examine time to all-cause mortality differences by duration of support stratification.

A 1:1 cardinality matching with a standardized difference tolerance set to 0.1 that matched on age, sex, race, and other tMCS prior to Impella 5.5 implant, and exact matching on etiology was evaluated. The outcomes of the matched population were compared between the duration of support stratification of DOS ≤14 days and DOS >14 days.

A p-value of <0.05 was considered statistically significant, and all reported p-values were two-tailed. All analyses were performed using R version 4.4.1 or SAS version 9.4 (SAS Institute Inc., Cary, NC, USA).

## Results

A total of 2262 patients were enrolled in the LOQI Registry, of which 443 patients had Impella 5.5 implants and were included in this analysis. Of the 443 patients, 306 were supported for DOS ≤14 days and 137 patients were supported for DOS >14 days (Supplemental Figure 1). Overall, the median age was 61 years (IQR: 53–68) and 19.4% were female. Patients had high rate of comorbidities such as diabetes (41.6%), hypertension (67.8%), coronary artery disease (55.3%), chronic kidney disease (29.3%), and prior PCI (28.3%), and among those with heart failure predominately were NYHA Class III/IV (86.2%) (Table 1). Patients in the DOS≤14 days were older (median: 62 vs. 57 years; p<0.001), female (23.2% vs. 10.9%; p=0.003) and had a higher comorbidity burden of hypertension (71.1% vs. 60.6%; p=0.03). Prior pre-admission history of heart failure treatment or heart failure hospitalization was higher in the DOS >14 days (65.7% vs. 52.1%; p=0.008) (Table 1). Severity of illness was stratified using a modified SCAI-CSWG stage based on pre-Impella 5.5 treatment intensity with 72% of the patients found to be SCAI stage C or above (Supplemental Table 2). Further breakdown by duration of support found higher proportion of patients in SCAI stage B-C (31.7% vs. 19.7%) and stage E (31.4% vs. 21.9%) in DOS ≤14 days compared to >14 days, and conversely a higher proportion of SCAI stage C-D (26.3% vs. 14.7%) and stage D (26.4% vs. 19.9%) in DOS >14 days compared to DOS ≤14 days (Supplemental Table 3).

**Table 1:** Baseline Demographics and Clinical Characteristics

Characteristics	All N = 443	DOS ≤14 Days N = 306	DOS >14 Days N = 137	P-Value^a^
Age, years	61.0 (53.0, 68.0) (443)	62.0 (55.0, 70.0) (306)	57.0 (48.0, 64.0) (137)	<0.001
Sex, Female	19.4% (86/443)	23.2% (71/306)	10.9% (15/137)	0.003
Race				0.004
White	69.3% (271/391)	74.6% (197/264)	58.3% (74/127)	
Black or African American	16.9% (66/391)	12.5% (33/264)	26.0% (33/127)	
Asian	5.6% (22/391)	4.9% (13/264)	7.1% (9/127)	
Other	8.2% (32/391)	8.0% (21/264)	8.7% (11/127)	
Body Mass Index (BMI), kg/m^2^	28.2 (24.9, 32.6) (419)	28.4 (25.0, 32.8) (288)	27.8 (24.7, 32.3) (131)	0.49
**Medical History and Comorbidities**				
Diabetes Mellitus	41.6% (184/442)	43.6% (133/305)	37.2% (51/137)	0.21
Hypertension	67.8% (299/441)	71.1% (216/304)	60.6% (83/137)	0.03
Coronary Artery Disease	55.3% (244/441)	57.2% (174/304)	51.1% (70/137)	0.23
Prior CVA/Stroke	13.4% (59/440)	14.2% (43/303)	11.7% (16/137)	0.47
Chronic Kidney Disease	29.3% (129/441)	28.3% (86/304)	31.4% (43/137)	0.51
Requiring Dialysis	24.2% (31/128)	23.3% (20/86)	26.2% (11/42)	0.72
Bleeding Disorder	3.9% (17/441)	3.6% (11/304)	4.4% (6/137)	0.70
Prior Myocardial Infarction (MI)	30.5% (134/440)	32.3% (98/303)	26.3% (36/137)	0.20
Prior Percutaneous Coronary Intervention (PCI)	28.3% (125/441)	30.3% (92/304)	24.1% (33/137)	0.18
Prior Coronary Artery Bypass Grafting (CABG)	9.8% (43/441)	9.2% (28/304)	10.9% (15/137)	0.57
Pre-admission history of treatments or hospital admissions for heart failure	56.4% (248/440)	52.1% (158/303)	65.7% (90/137)	0.008
HFrEF (≤40%)	89.5% (222/248)	88.0% (139/158)	92.2% (83/90)	—
HFpEF (>50%)	3.2% (8/248)	4.4% (7/158)	1.1% (1/90)	—
Unknown	7.3% (18/248)	7.6% (12/158)	6.7% (6/90)	—
NYHA Class within 180days prior to admission				0.46
I/II	13.8% (25/181)	15.3% (17/111)	11.4% (8/70)	
III/IV	86.2% (156/181)	84.7% (94/111)	88.6% (62/70)	
**Hemodynamics and Labs**				
LVEF (%)	20.0 (15.0, 30.0) (365)	20.0 (17.0, 30.0) (256)	20.0 (15.0, 25.0) (109)	0.003
Heart Rate (bpm)	92.0 (78.0, 107.0) (436)	90.5 (76.0, 106.0) (302)	94.5 (80.0, 109.0) (134)	0.078
SBP (mmHg)	108.0 (95.0, 124.0) (431)	110.0 (96.0, 129.0) (299)	104.0 (95.0, 114.0) (132)	0.002
DABP (mmHg)	73.0 (63.0, 84.0) (431)	73.0 (62.0, 86.0) (299)	73.0 (63.5, 80.0) (132)	0.55
MAP (mmHg)	85.0 (76.0, 96.0) (431)	86.0 (76.0, 99.0) (299)	84.0 (77.0, 91.0) (132)	0.068
Serum Creatinine (mg/dL)	1.4 (1.0, 1.9) (433)	1.3 (1.0, 1.8) (298)	1.5 (1.0, 2.0) (135)	0.055
eGFR (mL/min/1.73m^2^)	54.0 (37.0, 65.0) (377)	55.0 (38.0, 63.5) (261)	51.0 (35.9, 68.5) (116)	0.58
Hemoglobin (g/dL)	12.6 (10.9, 14.4) (430)	12.7 (10.9, 14.5) (297)	12.4 (10.8, 14.1) (133)	0.63
Lactate (mmol/L)	2.1 (1.4, 4.3) (217)	2.1 (1.4, 4.9) (139)	2.0 (1.4, 3.7) (78)	0.87
Platelets (10^3^/uL)	210.5 (161.0, 264.0) (430)	212.5 (165.0, 271.0) (294)	208.0 (155.0, 261.0) (136)	0.41
WBC (10^3^/uL)	9.2 (6.9, 13.9) (430)	9.3 (7.0, 13.8) (294)	9.1 (6.7, 13.9) (136)	0.59
**Context of Impella 5.5 Support**				
Received MCS prior to first Impella implant	34.5% (147/426)	31.6% (93/294)	40.9% (54/132)	0.06
ECMO + Other	39.5% (58/147)	38.7% (36/93)	40.7% (22/54)	—
Other	60.5% (89/147)	61.3% (57/93)	59.3% (32/54)	—
Cardiogenic shock prior to Impella device implant	80.4% (356/443)	75.8% (232/306)	90.5% (124/137)	<0.001
Cardiogenic Shock Setting				<0.001
AMICS	25.0% (89/356)	28.1% (65/231)	19.2% (24/125)	—
PCCS	6.7% (24/356)	10.0% (23/231)	0.8% (1/125)	—
HFCS	39.0% (139/356)	33.8% (78/231)	48.8% (61/125)	—
Myocarditis	0.8% (3/356)	0.0% (0/231)	2.4% (3/125)	—
Other	12.9% (46/356)	13.4% (31/231)	12.0% (15/125)	—
Unknown	15.4% (55/356)	14.7% (34/231)	16.8% (21/125)	—
**Duration (days)**				
Time from index admission to Impella 5.5	5 (2, 11) (443)	5 (2, 10) (306)	7 (3, 12) (137)	0.008
Duration of Impella 5.5 Support	9 (5, 17) (443)	7 (4, 10) (306)	23 (18, 33) (137)	<0.001
Total Cohort Support Duration	5884 days	2089 days	3795 days	—
Length of Hospital Stay	29 (18, 47) (442)	22 (15, 34) (305)	52 (38, 66) (137)	<0.001

Data summarized as either % (n/N), or median (Q1, Q3) n.

a p-values represent comparison between ≤ 14 days vs. > 14 days with either t-test, Wilcoxon rank sum test, fishers exact test, or Chi-square, as appropriate.

### Context of Impella 5.5 support

The Impella 5.5 devices were implanted at a median of 5 days (IQR: 2, 11) from index admission for the overall cohort with longer time to support for DOS >14 days compared to DOS ≤14 days (7 vs. 5 days; p=0.007). The overall median duration of support was 9 days (IQR: 5, 17), with 7 days (IQR: 4,10) and 23 days (IQR: 18, 33) for DOS ≤14 days and DOS >14 days cohorts, respectively. The overall median length of hospital stay was 29 days (IQR: 18, 47), with hospital stay being longer for the DOS >14 days (median: 52 vs. 22 days; p<0.001) compared to DOS ≤14 days (Table 1). Prior to receiving Impella 5.5, 147 patients were supported with other MCS devices, with ECMO usage accounting for 39.5% (n=58). There was a higher proportion of patients with other MCS devices prior to first Impella device in DOS >14 days (40.9% vs. 31.6%; p=0.06). Majority of the patients exhibited cardiogenic shock prior to implant (80.4%) with higher proportions in the DOS >14 days cohort (90.5% vs. 75.8%; p<0.001). Patients in the DOS >14 days had higher prevalence of HFCS (48.8% vs. 33.8%) and less AMICS (19.2% vs. 28.1%) (Table 1).

### Clinical outcomes and serious adverse events

Overall survival to discharge for the study cohort was 69%. Patients that survived were younger in age (median: 59 vs. 64 years; p<0.001) and had a lower incidence of history of bleeding disorders (1.6% vs. 8.8%; p<0.001), prior CABG (6.9% vs. 16.2%; p=0.002), and an MCS device prior to first Impella implant (26.4% vs. 52.6%; p<0.001), with a shock setting predominately being HFCS (45.5% vs. 26.8%) **(**Supplemental Table 4**)**. There was a significant difference in status at discharge between the two support durations (p<0.001) (Supplemental Table 5).

**Figure 1:**
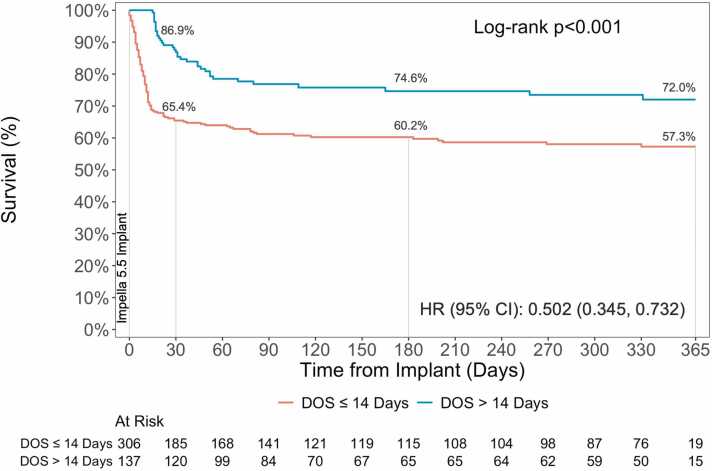
Survival from time of Impella 5.5 implant. Kaplan-Meier survival estimates through 1-year. P-values represent comparison between DOS ≤ 14 Days and > 14 Days.

**Figure 2:**
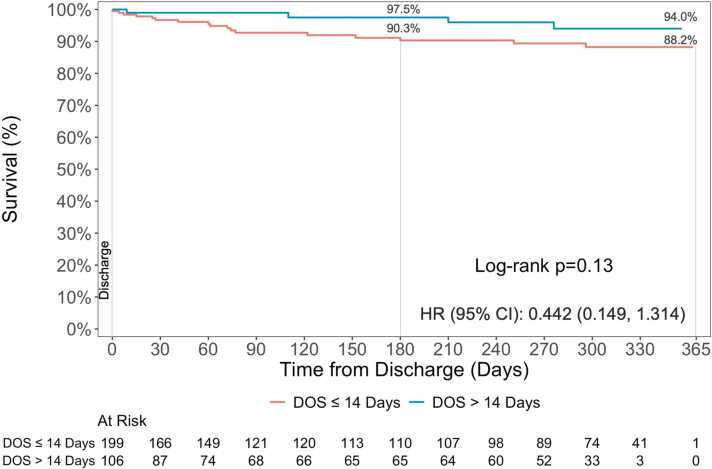
Freedom from All-Cause Mortality: Conditional Survival at 1-year. Kaplan-Meier survival estimates among patients that survived to discharge through 1-year. P-values represent comparison between DOS ≤ 14 Days and > 14 Days.

The one-year survival from Impella 5.5 implant was 61.7%. Per the inclusion criteria, the 30-day survival rate among patients in DOS >14 days cohort was higher compared to DOS ≤14 days (86.9% vs. 65.4%). This trend was observed through the 1-year post-implant (72.0% vs. 57.3%) reaching statistical significance (HR: 0.502, 95% CI: 0.342, 0.732; p<0.001) (Figure 1). Among patients that survived through discharge, numerically higher conditional survival rates were observed in the DOS >14 days group through 1-year (94.0% vs. 88.2%; HR: 0.442; 95% CI: 0.149, 1.314; p=0.13) (Figure 2). Further subgroup analysis demonstrated no difference in survival among those with an LVAD, heart transplant, or those without heart replacement therapies (Supplemental Figures 2 and 3). The 1-year survival post implant in patients that received heart transplant or LVAD was 95.5% compared to 86.0% in those with NHS (Supplemental Figure 4). The survival rates were consistent among patients that were discharged alive from the index hospitalization (HT/LVAD: 95.1%; NHS: 86.2%) (Supplemental Figure 5).

**Figure 3:**
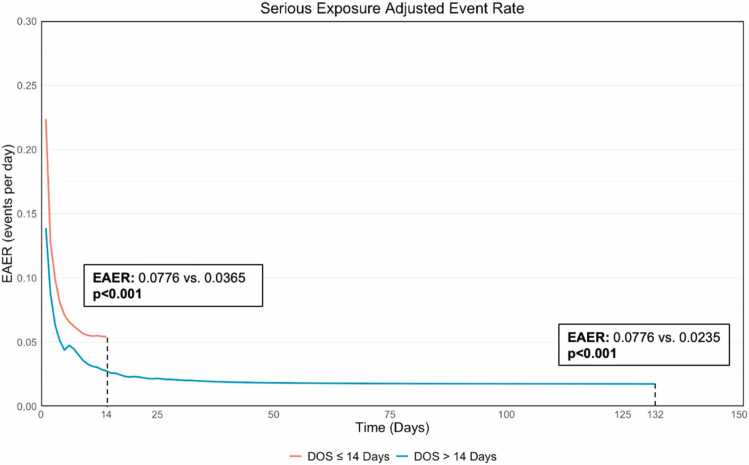
Cumulative Exposure Adjusted Event Rate (EAER) for all Serious Adverse Events while on Impella 5.5 support. All serious adverse events of interest (hemolysis, stroke, renal failure, and vascular complications) for the duration of support. Adverse event rate were compared using a Poisson regression model using the number of events as dependent variable and log(subject total duration of support). P-values represent comparison between DOS ≤ 14 Days and > 14 Days.

**Figure 4:**
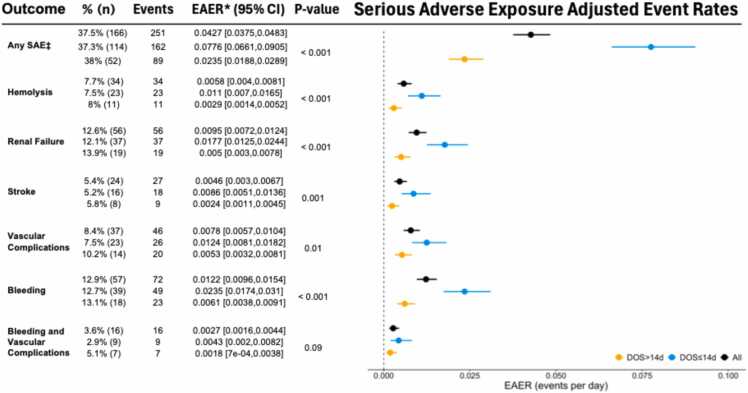
EAER for Serious Adverse Events Subtypes. Adverse event rate were compared using a Poisson regression model using the number of events as dependent variable and log(subject total duration of support). P-values represent comparison between DOS ≤ 14 Days and > 14 Days.

In the overall cohort, there were 251 serious adverse events (≥1 occurrence of stroke, hemolysis, renal failure, vascular complication, bleeding, or bleeding and vascular complications combined), which occurred in 37.5% of patients. The serious exposure adjusted event rate (EAER) for the overall cohort was 0.0427 (CI: 0.0375, 0.0483). The EAER curve was lower in the DOS >14 days through the first 14 days (0.0365 vs. 0.0776; p<0.001) and through the entire support duration (0.0235vs. 0.0776; p<0.001) (Figure 3, Figure 4). This trend was observed in hemolysis (0.0029 vs. 0.011), renal failure (0.005 vs. 0.0177), stroke (0.0024 vs. 0.0086), vascular complications (0.0053 vs. 0.0124), and bleeding (0.0061 vs. 0.0235) all reaching statistical significance (p<0.05) (Figure 4) demonstrating a lower adverse event profile in the DOS >14 days cohort. These findings were consistent for adverse events attributed to the device (Supplemental Figure 6).

### Matched analysis

**Figure 5:**
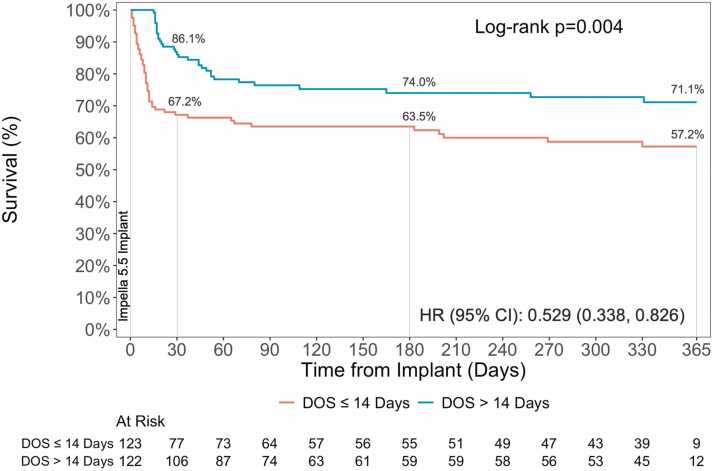
Survival from time of Impella 5.5 implant in Matched Cohort. Kaplan-Meier survival estimates through 1-year from implant in 245 matched cohort. P-values represent comparison between DOS ≤ 14 Days and > 14 Days.

**Figure 6:**
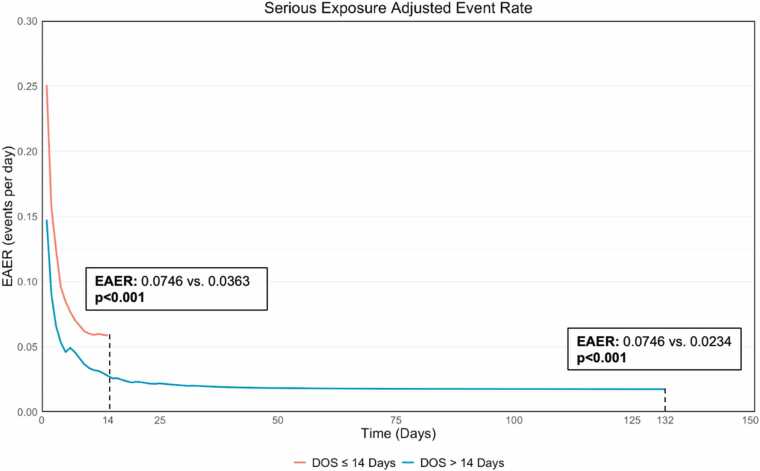
EAER for all Serious Adverse Events in Matched Cohort. Adverse event rate were compared using a Poisson regression model using the number of events as dependent variable and log(subject total duration of support) in 245 matched patients. P-values represent comparison between DOS ≤ 14 Days and > 14 Days.

In an analysis matching based on age, sex, race, previous MCS, and an exact match on etiology yielded a robust patient cohort that was well below the identified absolute standardized difference cut-off (Supplemental Figure 7). A total of 245 patients were matched to the two groups, the ≤14 days cohort included 123 patients, and the >14 days cohort included 122 patients. The baseline characteristics and medical history were similar between the two groups with no statistical differences observed (Table 2). Survival from implant was higher in the DOS >14 days cohort (71.1% vs. 57.2%; HR: 0.529; 95% CI: 0.338, 0.826; p=0.004) through 1 year, with no difference among patients who survived from discharge (93.4% vs. 86.2%; HR: 0.467; 95% CI: 0.140, 1.551; p=0.20) (Figure 5, Supplemental Figure 8). In the matched cohort, the <14 day cohort had more patients with NHS (37.4% vs. 32.0%) and death (33.3% vs. 23.0%), with less heart transplants (13.8% vs. 34.4%) compared to >14 days (Supplemental Table 6). As observed in the matched patients, the serious exposure adjusted event rate was lower in the DOS >14 days as compared to DOS ≤14 days (0.0234 vs. 0.0746; p<0.001). This was consistent in hemolysis (0.0033 vs. 0.0119; p=0.004), renal failure (0.0045 vs. 0.0184; p<0.001), stroke (0.0024 vs. 0.0108; p=0.002), vascular complications (0.0051vs. 0.013; p=0.02), and bleeding (0.006 vs. 0.0162; p=0.005) (Figure 6, Figure Supplemental 9).

**Table 2:** Baseline Demographics and Clinical Characteristics of Matched Cohort

**Characteristics**	**All N = 245**	**DOS ≤ 14 Days N = 123**	**DOS > 14 Days N = 122**	**P-Value**^a^
Age (years)	58.0 (50.0, 64.0) (245)	58.0 (51.0, 64.0) (123)	58.0 (49.0, 65.0) (122)	0.76
Sex Female	12.7% (31/245)	13.8% (17/123)	11.5% (14/122)	0.58
Race				0.97
White	65.3% (147/225)	65.5% (74/113)	65.2% (73/112)	
Black	21.3% (48/225)	20.4% (23/113)	22.3% (25/112)	
Asian	4.9% (11/225)	5.3% (6/113)	4.5% (5/112)	
Other	8.4% (19/225)	8.8% (10/113)	8.0% (9/112)	
BMI (kg/m^2^)	28.0 (24.8, 32.1) (237)	28.3 (24.8, 32.2) (119)	27.8 (24.7, 32.1) (118)	0.94
**Medical History and Comorbidities**				
NYHA Class				0.24
I/II	14.8% (18/122)	18.6% (11/59)	11.1% (7/63)	
III/IV	85.2% (104/122)	81.4% (48/59)	88.9% (56/63)	
Diabetes Mellitus	37.6% (92/245)	37.4% (46/123)	37.7% (46/122)	0.96
Hypertension	64.5% (158/245)	67.5% (83/123)	61.5% (75/122)	0.33
Coronary Artery Disease	53.9% (132/245)	54.5% (67/123)	53.3% (65/122)	0.85
Prior CVA/Stroke	13.9% (34/245)	17.1% (21/123)	10.7% (13/122)	0.15
Chronic Kidney Disease	33.5% (82/245)	39.0% (48/123)	27.9% (34/122)	0.064
Requiring Dialysis	24.7% (20/81)	20.8% (10/48)	30.3% (10/33)	0.33
History of Bleeding Disorder	4.5% (11/245)	4.9% (6/123)	4.1% (5/122)	0.77
Prior Myocardial Infarction (MI)	28.6% (70/245)	30.1% (37/123)	27.0% (33/122)	0.6
Prior Percutaneous Coronary Intervention (PCI)	24.9% (61/245)	26.0% (32/123)	23.8% (29/122)	0.68
Prior Coronary Artery Bypass Grafting (CABG)	10.6% (26/245)	9.8% (12/123)	11.5% (14/122)	0.66
Pre-admission history of treatments or hospital admissions for heart failure	62.9% (154/245)	61.8% (76/123)	63.9% (78/122)	0.73
HFrEF (≤40%)	91.6% (141/154)	88.2% (67/76)	94.9% (74/78)	
HFpEF (>50%)	1.9% (3/154)	2.6% (2/76)	1.3% (1/78)	
Unknown	6.5% (10/154)	9.2% (7/76)	3.8% (3/78)	
**Hemodynamics and Labs**				
LVEF (%)	20.0 (15.0, 25.0) (204)	20.0 (15.0, 25.0) (107)	20.0 (14.0, 25.0) (97)	0.46
Heart Rate (bpm)	95.0 (80.0, 109.0) (242)	95.0 (80.0, 110.0) (123)	95.0 (80.0, 109.0) (119)	0.6
SBP (mmHg)	105.0 (94.0, 118.0) (238)	107.0 (94.0, 124.0) (121)	103.0 (95.0, 114.0) (117)	0.15
DABP (mmHg)	73.0 (63.0, 82.0) (238)	73.0 (63.0, 84.0) (121)	73.0 (63.0, 81.0) (117)	0.77
MAP (mmHg)	84.0 (76.0, 93.0) (238)	85.0 (76.0, 98.0) (121)	84.0 (76.0, 91.0) (117)	0.28
Serum Creatinine (mg/dL)	1.5 (1.1, 2.1) (243)	1.5 (1.1, 2.1) (122)	1.5 (1.0, 2.0) (121)	0.75
eGFR (mL/min/1.73m^2)	51.0 (36.0, 63.7) (213)	51.5 (37.0, 61.0) (108)	51.0 (35.7, 69.0) (105)	0.74
Hemoglobin (g/dL)	12.7 (10.9, 14.2) (238)	12.9 (11.2, 14.6) (120)	12.5 (10.8, 14.0) (118)	0.29
Lactate (mmol/L)	2.0 (1.4, 3.8) (133)	2.3 (1.4, 5.1) (62)	2.0 (1.3, 3.7) (71)	0.72
Platelets (10^3/uL)	200.0 (155.0, 263.5) (240)	199.0 (159.0, 272.0) (119)	204.0 (152.0, 259.0) (121)	0.49
WBC (10^3/uL)	9.4 (6.7, 14.0) (240)	9.5 (6.7, 14.5) (119)	9.1 (6.8, 13.9) (121)	0.63
**Context of Impella 5.5 Support**				
Received MCS prior to first Impella implant	38.8% (92/237)	37.0% (44/119)	40.7% (48/118)	0.56
ECMO + Other	39.1% (36/92)	36.4% (16/44)	41.7% (20/48)	—
Other	60.9% (56/92)	63.6% (28/44)	58.3% (28/48)	—
Cardiogenic shock prior to Impella device implant	90.2% (231/256)	90.6% (116/128)	89.8% (115/128)	0.83
Cardiogenic Shock Setting				>0.99
AMICS	20.8% (46/221)	20.7% (23/111)	20.9% (23/110)	
HFCS	52.5% (116/221)	52.3% (58/111)	52.7% (58/110)	
Other	10.0% (22/221)	9.9% (11/111)	10.0% (11/110)	
Unknown	16.7% (37/221)	17.1% (19/111)	16.4% (18/110)	
**Duration**				
Time from index admission to Impella 5.5 (days)	6 (2, 11) (245)	6 (1, 10) (123)	7 (2, 12) (122)	0.11
Duration of Impella 5.5 Support (days)	14 (8, 22) (245)	8 (5, 11) (123)	23 (18, 32) (122)	<0.001
Total Cohort Support Duration (days)	4257.01	924.51	3332.50	—
Length of Hospital Stay (days)	35 (22, 57) (245)	23 (15, 35) (123)	51 (36, 65) (122)	<0.001

Data summarized as either % (n/N), or median (Q1, Q3) n.

a p-values represent comparison between ≤ 14 days vs. > 14 days with either t-test, Wilcoxon rank sum test, fishers exact test, or Chi-square, as appropriate.

## Discussion

The Impella 5.5 microaxial pump has seen increasing adoption for the treatment of cardiogenic shock.^9^ Real world clinical practice has demonstrated that a subset of patients presenting in cardiogenic shock require longer durations of support to achieve hemodynamic stability, reversal of end-organ dysfunction, and rehabilitation.^8^, ^10^, ^11^, ^12^, ^13^ Extended duration support for some patients has also allowed time to assess for the potential of native heart survival, avoiding premature transition to heart replacement therapies. It is also an effective bridge to heart transplant or LVAD placement for patients who may have prolonged wait times or other medical or social concerns requiring additional time on support. While FDA-approved for 14 days, multiple observational studies have demonstrated excellent outcomes in patients supported for >14 days on Impella 5.5.^1^, ^3^ In this large multi-center registry-based analysis of patients implanted with Impella 5.5 we performed a unique analysis with long term clinical outcomes and adverse events in patients receiving short or long duration of support with the Impella 5.5. Compared to prior published observational, retrospective studies, this registry includes a more heterogenous patient population, center variety, and long-term outcome assessment.

Over the entire cohort, there was a high severity of illness, with 72% of patients in SCAI stage C or above and 39.5% of patients requiring VA ECMO. One third of all patients received an MCS device prior to Impella implant. AMI-CS was more common in the DOS ≤14 days group, with HFCS being more common in the >14 day DOS group. Notably, all but one patient in the post-cardiotomy CS group had DOS <14 days.

Overall survival to discharge for the study cohort was 69.0%. Higher survival rates to hospital discharge were seen in those supported >14 days compared to ≤14 days (77.4% vs 65.2% respectively). From the time of device implantation, one year survival was 70% in DOS >14 days compared to 57.3% in ≤14 days, with curves separating early. The conditional survival in patients discharged from the hospital (with either NHS or HRT) was similar between cohorts at 180 days. In patients surviving to discharge, fewer patients received MCS prior to Impella (26.4% vs 52.6%) compared to those who expired prior to discharge. It should be acknowledged that the survival differential between cohorts can be explained by several factors. In this sick cohort of patients, some patients exhibited early mortality during an early hazard phase within the first 14 days and therefore do not progress to support beyond 14 days. In addition, the etiology of shock was different in the two groups, with more AMI-CS patients in the shorter duration cohort, and a greater percentage of patients with HF-CS in the long duration cohort. Previous studies have demonstrated a higher overall mortality for AMI-CS when compared to HF-CS.^14^ Also, a greater percentage of patients in the long duration cohort underwent transition to durable replacement therapies with transplant (35% vs. 8.9%) or LVAD (10.9% vs. 8.9%), further contributing to improved long-term outcomes in patients with extended duration support.

To account for the differences in duration of support and assess the incidence of adverse events indexed to units of time between the two groups, an exposure adjusted event rate (EAER) analysis was performed. Adverse event rates were consistent with previously reported adverse event rates in the published literature.^3^ Of the total cohort, any SAE occurred in 37.5% of patients. When comparing short versus long duration support, the EAER rate was lower in the >14 day group compared to the ≤14 day group for all SAEs including hemolysis, renal failure, stroke and vascular complications. These data demonstrate that there was no incremental increase in adverse event rates associated with Impella 5.5 support beyond 14 days, also highlighting that the majority of adverse events occurred in the early acute phase of care. In the propensity-matched cohort, we similarly observed no increase in mortality or exposure-adjusted adverse event rates with extended duration support; however, we note that the absolute 14-day event comparison remained higher in the ≤14-day cohort despite matching, consistent with an early hazard phase and likely residual unmeasured confounding and selection effects inherent to defining a >14-day cohort.

Extended duration of support for a subset of patients suffering from cardiogenic shock allowed additional time to achieve NHS and safe bridging to durable replacement therapies. With respect to recovery, NHS was achieved in 42.5%. There were 47.5% of patients achieving NHS to explant in the DOS ≤14 days group, and 31.4% in the >14 days group. Therefore, almost one third of patients who were supported beyond 14 days demonstrated sufficient recovery of native cardiac function to allow for device explant without the need for replacement therapies. Comparatively, a large CSWG analysis recently published by Kanwar et. al. also reported NHS was more frequent in DOS ≤14 (29.8%) compared to DOS >14 days (21.8%).^3^ NHS was more common in this registry, which will need further analysis for better understanding, but may be partially explained by a higher percentage of post-cardiotomy shock patients in this cohort, or practice variability across centers. Specifically, early HRT vs prolonged duration with attempts at GDMT and NHS in subgroups of patients. For example, in a single-center study assessing outcomes in CS patients supported with Impella 5.0/5.5, Genstler et.al confirmed improved NHS in patients receiving guideline directed medical therapy (GDMT).^15^

One year survival in patients discharged with NHS was excellent, at 86.2%. Variations in rates of NHS in the published literature highlight the opportunity in the community to focus on recent best practices and medical management. Overall, these data are encouraging for Impella 5.5 use as bridge to NHS. Several publications also re-- the initiation of GDMT to promote heart recovery on Impella devices.^12^, ^16^ While GDMT use was not assessed in this analysis, several ongoing studies are evaluating the potential synergies of temporary MCS and GDMT to promote myocardial recovery. Other compelling data include recent work by Bandini et. al demonstrating 54% NHS in patients receiving Impella 5/5.5 for cardiogenic shock utilizing strict hemodynamic monitoring with PA catheters and use of GDMT during weaning.^17^ The majority of those patients were AMI-CS (60%) and SCAI D or E (65%). At 180 days survival was 95% with zero patients requiring HRT after discharge.

Regional variations in donor availability can lead to unpredictable and prolonged waiting times for heart transplantation. Total bridging to HRT occurred in just over 26.5% of patients. While 75 patients of the total cohort were bridged to heart transplantation, only 27 patients (36%) were transplanted in ≤14 days. Organ Procurement and Transplantation Network (OPTN) data show increasing numbers of patients wait-listed for transplant.^18^ While strategies for increasing available donor hearts have been developed (DCD, OCS devices), demand for donor hearts far exceeds the supply of organs. In this cohort, extended duration support was necessary in nearly two thirds of patients awaiting heart transplantation.

## Limitations

This analysis reports several limitations. First limitation of this analysis includes the realization that patients that die early are less likely to accrue 14 days of support on device and will not have further events introducing potential survivorship bias. Second, the LOQI registry does not capture details of patient’s course prior to enrollment and during support, which includes data that could impact outcomes. Such events include complications associated with right heart failure and infection – although clinically important in temporary MCS – could not be evaluated in the present analysis due to data not being captured with sufficient granularity. Third, the NHS population includes patients that may include those that transitioned to palliative therapies. Finally, despite matching, clinically unmeasured confounding likely persists between the short and extended-duration cohorts. Despite this limitation, these results are promising for safety and efficacy of utilizing Impella 5.5 as a bridge to HRT and NHS.

## Conclusion

Extended duration of support with the Impella 5.5 has emerged as an important treatment option for a subset of patients presenting in cardiogenic shock, both to achieve NHS and to safely bridge patients to HRT with transplantation or durable LVAD placement. Utilizing EAER and propensity matched analysis, there were lower exposure adjusted event rates in those supported >14 days, supporting the hypothesis that support beyond 14 days did not expose patients to an increased incremental risk of adverse events. Despite a high severity of illness, nearly half of patients were able to achieve NHS with device explant. Future research in Impella 5.5 utilization in cardiogenic shock should focus on use of best practices, with a focus on reducing SAEs, and optimizing GDMT in conjunction with temporary mechanical support to potentially increase rates of NHS.

## Financial Disclosures

David Kaczorowski: Consultant – Johnson&Johnson MedTech; Research support – Transmedics, XVIVO

Vidang P Nguyen: Consultant – Johnson&Johnson MedTech

Aasim Afzal: Consultant Abiomed, Abbott, Bridge Bio, Alnylam

Scott C Silvestry: Consultant – Abbott, Johnson&Johnson MedTech, Medtronic, Syncardia

Ahmad Zeeshan: Consultant – Johnson&Johnson MedTech

Naveena Yanamala: Consultant/Advisor: Magnetic 3D, Research Spark Hub, Turnkey Learning & Turnkey TechStart (I) Pvt. Ltd; Research Support – Johnson&Johnson MedTech, Novara Solutions Group LLC.

Ray Matthews: Speaker and Research Support – Johnson&Johnson MedTech

Rothy Chhim: Employee of Johnson&Johnson MedTech

Sanjeev Aggarwal: Employee of Johnson&Johnson MedTech

Sami Somo: Employee of Johnson&Johnson MedTech

Gavin W Hickey: Consultant – Johnson&Johnson MedTech

## Declaration of Competing Interest

The authors declare the following financial interests/personal relationships which may be considered as potential competing interests: Gavin W Hickey reports article publishing charges, and statistical analysis provided by Johnson & Johnson MedTech. David Kaczorowski reports a relationship with Johnson & Johnson MedTech that includes: consulting or advisory. David Kaczorowski reports a relationship with TransMedics Inc that includes: consulting or advisory. Vidang Nguyen reports a relationship with Johnson & Johnson MedTech that includes: consulting or advisory. Aasim Afzal reports a relationship with AbioMed Inc that includes: consulting or advisory. Aasim Afzal reports a relationship with Abbott that includes: consulting or advisory. Aasim Afzal reports a relationship with Bridge Biotherapeutics, Inc that includes: consulting or advisory. Aasim Afzal reports a relationship with Alnylam Pharmaceuticals Inc that includes: consulting or advisory. Scott Silvestry reports a relationship with Abbott that includes: consulting or advisory. Scott Silvestry reports a relationship with Johnson & Johnson MedTech that includes: consulting or advisory. Scott Silvestry reports a relationship with Medtronic that includes: consulting or advisory. Scott Silvestry reports a relationship with SynCardia Systems Llc that includes: consulting or advisory. Naveena Yanamala reports a relationship with Turnkey Opportunities Inc that includes: consulting or advisory. Naveena Yanamala reports a relationship with Johnson & Johnson MedTech that includes: consulting or advisory. Naveena Yanamala reports a relationship with Novara solutions group LLC that includes: funding grants. Ray Matthews reports a relationship with Johnson & Johnson MedTech that includes: speaking and lecture fees. Rothy Chinn reports a relationship with Johnson & Johnson MedTech that includes: employment. Sanjeev Aggarwal reports a relationship with Johnson & Johnson MedTech that includes: employment. Sami Somo reports a relationship with Johnson & Johnson MedTech that includes: employment. If there are other authors, they declare that they have no known competing financial interests or personal relationships that could have appeared to influence the work reported in this paper.

## Footnotes

Appendix A Supplemental data associated with this article can be found in the online version at doi:10.1016/j.jhlto.2026.100586.
