# Supplementary material for: Incidence and clinical outcomes of extended duration support in patients with Impella 5.5 - Analysis from the LOQI Registry
Source: JHLT Open. 2026 May 6;13:100586. doi: 10.1016/j.jhlto.2026.100586 (PMC13234711; doi:10.1016/j.jhlto.2026.100586)
Supplement: Supplementary file 1 — Supplementary material [file mmc1.docx]

**SUPPLEMENTAL MATERIAL**

This appendix has been provided by the authors to provide additional detail about their work.

Supplement to: David Kaczorowski et al. Incidence and clinical outcomes of extended duration support in patients with Impella 5.5 - Analysis from the LOQI Registry

**Supplemental Table 1. Adverse Event Definitions**

| **Renal Failure** | **Acute Kidney Injury (AKI)**  *Modified KDIGO*   \| Stage \| Criteria \| \| --- \| --- \| \| Stage 1 \| Increase in SCr by ≥ 0.3 mg/dL within 48 hours or increase in SCr 1.5 to 1.9 times baseline \| \| Stage 2 \| Increase in SCr to 2.0 to < 3.0 times baseline \| \| Stage 3 \| Increase in SCr to ≥ 3.0 times baseline or increase in SCr to ≥ 4.0 mg/dL or initiation of renal replacement therapy \|   Index procedure-related AKI will be classified as occurring within seven (7) days after the index procedure per definitions above. For the purposes of this definition, index procedure includes both the index PCI and any planned staged PCI procedures. Also, in-hospital is defined as both the index procedure and any planned staged PCI procedure (as applicable) |
| --- | --- | --- | --- | --- | --- | --- | --- | --- | --- |
| **Hemolysis** | Clinically relevant hemolysis based on institution criteria which may include increased serum LDH (i.e., >1.5x), plasma-free hemoglobin (pfHb) (i.e., >40mg/dl), elevated indirect bilirubin, new hemoglobinuria, anemia requiring transfusion (that is not attributed to another cause) and/or requiring device removal. |
| **Stroke** | **Stroke Diagnostic Criteria:**   - Rapid onset of a focal or global neurological deficit with at least one of the following: change in level of consciousness, hemiplegia, hemiparesis, numbness or sensory loss affecting one side of the body, dysphasia or aphasia, hemianopia, amaurosis fugax or other neurological signs or symptoms consistent with stroke - Duration of a focal or global neurological deficit ≥24 hours; OR <24 hours, if therapeutic intervention(s) were performed (e.g., thrombolytic therapy or intracranial angioplasty); OR available neuroimaging documents a new hemorrhage or infarct; OR the neurologic deficit results in death - No other readily identifiable non-stroke cause for the clinical presentation (e.g., brain tumor, trauma, infection, hypoglycemia, peripheral lesion, pharmacological influences) - Confirmation of the diagnosis by at least one of the following:   - Neurology or neurosurgical specialist   - Neuroimaging procedure (MRI or CT scan or cerebral angiography)   - Lumbar puncture (i.e., spinal fluid analysis diagnostic of intracranial hemorrhage)   **TIA Diagnostic Criteria:**   - Brief episode of neurologic dysfunction, caused by focal brain or retinal ischemia, with clinical symptoms typically less than one hour and without evidence of acute infarction   **Definition of TIA versus Stroke Syndromes:**   - TIA: New focal neurologic deficit with rapid symptoms resolution (usually 1-2 hours), always within <24 hours; neuroimaging always negative for acute tissue injury - Stroke: Diagnosis as above, preferably with positive neuroimaging study   Subjects with non-focal global encephalopathy will not be reported as stroke without unequivocal evidence based upon neuroimaging studies |
| **Vascular Complications** | *As defined by VARC-3 as Major Vascular Complication.*  Any one (1) of the following:   - Aortic dissection or aortic rupture - Vascular (arterial or venous) injury (perforation, rupture, dissection, stenosis, ischemia, arterial or venous thrombosis including pulmonary embolism, arteriovenous fistula, pseudoaneurysm, hematoma, retroperitoneal hematoma, infection) or compartment syndrome resulting in death, VARC Type ≥2 bleeding, limb or visceral ischemia, or irreversible neurologic impairment - Distal embolization (non-cerebral) from a vascular source resulting in death, amputation, limb or visceral ischemia, or irreversible neurologic impairment - Closure device failure* resulting in death, VARC Type ≥2 bleeding, limb or visceral ischemia, or irreversible neurologic impairment   * Any device-related vascular access site and any other accessory access sites (venous or arterial) used during procedure.  *NOTE:* Per VARC-3 Bleeding definition, VARC Type 2 bleeding correlates with BARC Type 3a. |
| **Bleeding, BARC ≥ 3** | Bleeding as defined by BARC^1^   1. Type 3: Clinical, laboratory and/or imaging evidence of bleeding with specific healthcare provider responses, as listed below:    - Type 3a:      - Any transfusion with overt bleeding      - Overt bleeding plus hemoglobin drop of 3 to 5 g/dL* (provided hemoglobin drop is related to bleeding).    - Type 3b:      - Overt bleeding plus hemoglobin drop ≥5 g/dL* (provided hemoglobin drop is related to bleed)      - Cardiac tamponade      - Bleeding requiring surgical intervention for control (excluding dental/nasal/skin/hemorrhoid)      - Bleeding requiring intravenous vasoactive agents    - Type 3c:      - Intracranial hemorrhage (does not include microbleeds or hemorrhagic transformation, does include intraspinal); subcategories confirmed by autopsy or imaging or lumbar puncture      - Intraocular bleed compromising vision 2. Type 4: Coronary artery bypass graft (CABG)-related bleeding  - Perioperative intracranial bleeding within 48 hours - Reoperation after closure of sternotomy for the purpose of controlling bleeding - Transfusion of ≥5 U whole blood or packed red blood cells within a 48-hour period (only allogeneic transfusions are considered transfusions for CABG-related bleeds) - Chest tube output >2L within a 24-hour period  1. Type 5: Fatal bleeding    - Type 5a: Probable      - Clinically suspicious as the cause of death but the bleeding is not directly observed and there is no autopsy or confirmatory imaging    - Type 5b: Definite      - Bleeding that is directly observed (by either clinical specimen or imaging) or confirmed on autopsy   ** Corrected for transfusion (1 unit packed red blood cells or 1 unit whole blood = 1 g/dL hemoglobin)*  *^1^Mehran R, Rao SV, Bhatt DL, et al. Standardized bleeding definitions for cardiovascular clinical trials: a consensus report from the Bleeding Academic Research Consortium. Circulation. 2011;123:2736-2747.* |

**Supplemental Figure 1.** Consort Diagram from the Long-Term Outcome and Quality Indicator Impella Registry (LOQI) Observational Registry


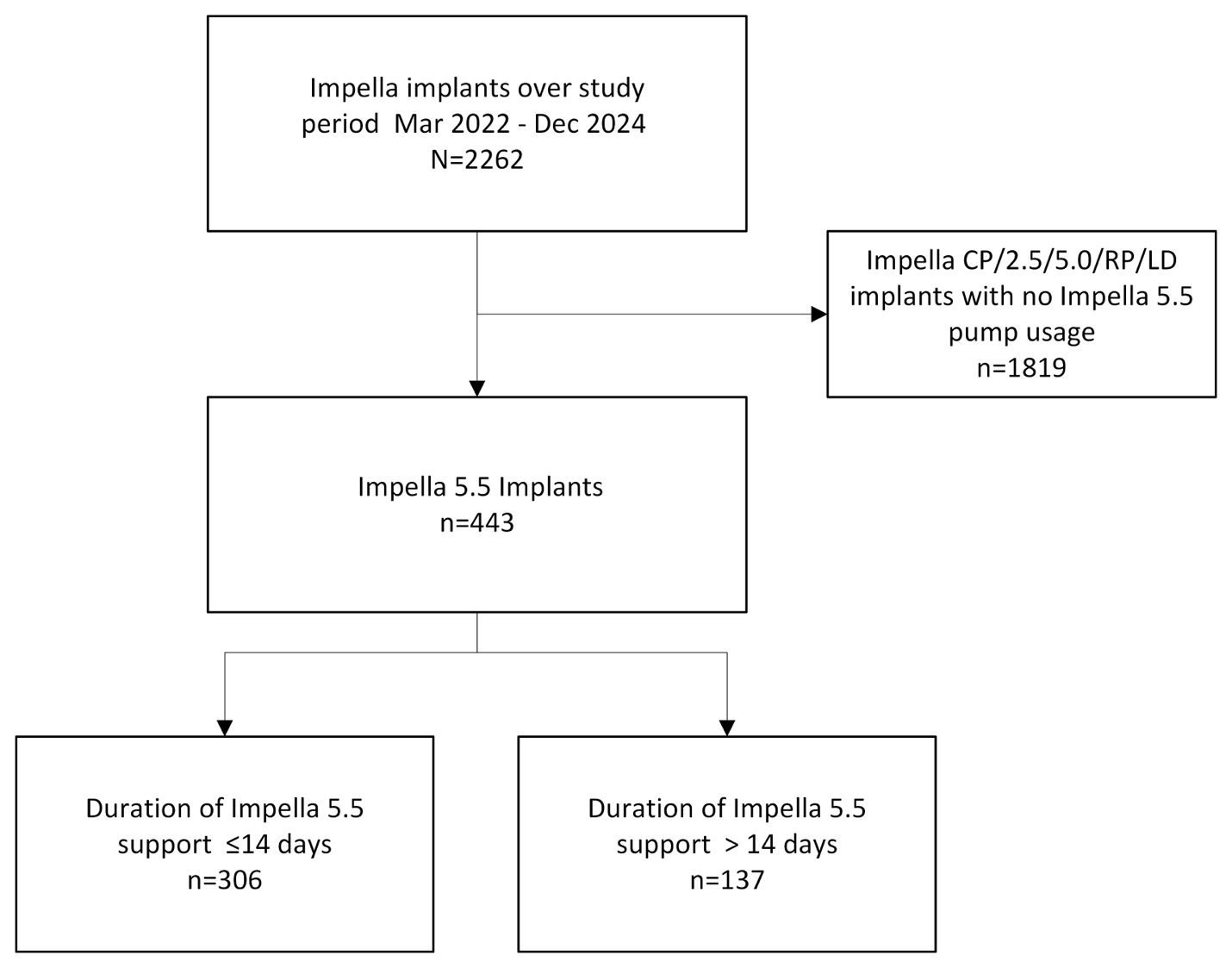


**Supplemental Table 2.** Categorization of patient severity using modified SCAI-CSWG stage based on pre-Impella treatment intensity

| **Characteristics** | **B-C** | **C** | **C-D** | **D** | **D-E** | **E** |
| --- | --- | --- | --- | --- | --- | --- |
| No MCS, No Drugs | 124/443 (28.0%) |  |  |  |  |  |
| VA-ECMO use prior to Impella 5.5 + 0 Drug |  | 0/443 (0%) |  |  |  |  |
| MCS (1 device) + No Drug |  |  | 24/443 (5.42%) |  |  |  |
| No MCS + 1 Drug |  |  | 57/443 (12.9%) |  |  |  |
| 2 MCS + 0 Drug |  |  |  | 3/443 (0.7%) |  |  |
| 0-2 MCS and 1-2 Drugs |  |  |  | 94/443 (21.2%) |  |  |
| 1-2 MCS + Only 1 Drug |  |  |  | 23/94 (24.5%) |  |  |
| 1-2 MCS + 2 Drug |  |  |  | 22/94 (23.4%) |  |  |
| No MCS + 2 Drug |  |  |  | 49/94 (52.1%) |  |  |
| VA-ECMO use prior to Impella 5.5 + ≥ 1 Drug |  |  |  |  | 13/443 (2.9%) |  |
| Cardiac Arrest Out of Hospital |  |  |  |  |  | 44/443 (9.9%) |
| VA-ECMO prior to Impella 5.5 + 0 Drug + Out of Hospital Arrest |  |  |  |  |  | 2/443 (0.5%) |
| ≥ 3 Drugs |  |  |  |  |  | 101/443 (22.8%) |
| ≥ 3 MCS Prior to Support |  |  |  |  |  | 0/443 (0%) |
| Total | 124/443 (28.0%) | 0/443 (0%) | 81/443 (18.3%) | 97/443 (21.9%) | 13/443 (2.9%) | 128/443 (28.9%) |

Abbreviations: SCAI, Society for Cardiovascular Angiography and Interventions; CSWG, Cardiogenic Shock Working Group; MCS, Mechanical Circulatory Support; Drugs = Inotropes and Vasopressors

**Supplemental Table 3. Categorization of patient severity using modified SCAI-CSWG stage based on pre-Impella treatment intensity: for DOS ≤14 and >14 days**

|  | All  N = 443 | DOS ≤ 14 Days  N = 306 | DOS > 14 Days  N = 137 | P-Value* |
| --- | --- | --- | --- | --- |
| Derived SCAI Classificatio**n** |  |  |  | 0.002 |
| B-C | 28.0% (124/443) | 31.7% (97/306) | 19.7% (27/137) |  |
| C | 0.0% (0/443) | 0.0% (0/306) | 0.0% (0/137) |  |
| C-D | 18.3% (81/443) | 14.7% (45/306) | 26.3% (36/137) |  |
| D | 21.9% (97/443) | 19.9% (61/306) | 26.3% (36/137) |  |
| D-E | 2.9% (13/443) | 2.3% (7/306) | 4.4% (6/137) |  |
| E | 28.9% (128/443) | 31.4% (96/306) | 23.4% (32/137) |  |

*p-values represent comparison between ≤ 14 days vs. > 14 days with either t-test, Wilcoxon rank sum test, fishers exact test, or Chi-square, as appropriate. Abbreviations: SCAI, Society for Cardiovascular Angiography and Interventions; CSWG, Cardiogenic Shock Working Group; MCS, Mechanical Circulatory Support; Drugs = Inotropes and Vasopressors

**Supplemental Table 4. Baseline Demographics and Clinical Characteristics of patients: Stratified by discharge outcome of Alive vs. Expired**

| Characteristics | All  N = 442 | Alive  N = 305 | Expired  N = 137 | P-Value* |
| --- | --- | --- | --- | --- |
| Age (years) | 61.0 (53.0, 68.0) (442) | 59.0 (51.0, 67.0) (305) | 64.0 (58.0, 70.0) (137) | <0.001 |
| Sex Female | 19.5% (86/442) | 17.7% (54/305) | 23.4% (32/137) | 0.17 |
| Race |  |  |  | 0.49 |
| White | 69.2% (270/390) | 66.8% (179/268) | 74.6% (91/122) |  |
| Black | 16.9% (66/390) | 18.3% (49/268) | 13.9% (17/122) |  |
| Asian | 5.6% (22/390) | 6.0% (16/268) | 4.9% (6/122) |  |
| Other | 8.2% (32/390) | 9.0% (24/268) | 6.6% (8/122) |  |
| BMI (kg/m^2^) | 28.2 (24.9, 32.6) (418) | 27.8 (24.5, 32.0) (291) | 29.4 (25.5, 33.2) (127) | 0.046 |
| **Medical History and Comorbidities** |  |  |  |  |
| NYHA Class |  |  |  | 0.17 |
| I/II | 13.8% (25/181) | 12.0% (17/142) | 20.5% (8/39) |  |
| III/IV | 86.2% (156/181) | 88.0% (125/142) | 79.5% (31/39) |  |
| Diabetes Mellitus | 41.7% (184/441) | 42.1% (128/304) | 40.9% (56/137) | 0.81 |
| Hypertension | 68.0% (299/440) | 66.4% (202/304) | 71.3% (97/136) | 0.31 |
| Coronary Artery Disease | 55.5% (244/440) | 53.6% (163/304) | 59.6% (81/136) | 0.25 |
| Prior CVA/Stroke | 13.4% (59/439) | 12.8% (39/304) | 14.8% (20/135) | 0.57 |
| Chronic Kidney Disease | 29.3% (129/440) | 30.3% (92/304) | 27.2% (37/136) | 0.52 |
| Requiring Dialysis | 24.2% (31/128) | 22.0% (20/91) | 29.7% (11/37) | 0.35 |
| History of Bleeding Disorder | 3.9% (17/440) | 1.6% (5/304) | 8.8% (12/136) | <0.001 |
| Prior Myocardial Infarction (MI) | 30.3% (133/439) | 29.7% (90/303) | 31.6% (43/136) | 0.69 |
| Prior Percutaneous Coronary Intervention (PCI) | 28.2% (124/440) | 27.0% (82/304) | 30.9% (42/136) | 0.4 |
| Prior Coronary Artery Bypass Grafting (CABG) | 9.8% (43/440) | 6.9% (21/304) | 16.2% (22/136) | 0.002 |
| Pre-admission history of treatments or hospital admissions for heart failure | 56.5% (248/439) | 62.8% (191/304) | 42.2% (57/135) | <0.001 |
| HFrEF (≤40%) | 89.5% (222/248) | 91.6% (175/191) | 82.5% (47/57) |  |
| HFpEF (>50%) | 3.2% (8/248) | 2.6% (5/191) | 5.3% (3/57) |  |
| Unknown | 7.3% (18/248) | 5.8% (11/191) | 12.3% (7/57) |  |
| **Hemodynamics and Labs** |  |  |  |  |
| LVEF (%) | 20.0 (15.0, 30.0) (364) | 20.0 (15.0, 26.0) (251) | 25.0 (18.6, 35.0) (113) | 0.006 |
| Heart Rate (bpm) | 92.0 (78.0, 107.0) (435) | 92.0 (80.0, 107.0) (300) | 90.0 (72.0, 109.0) (135) | 0.5 |
| SBP (mmHg) | 108.0 (95.0, 124.0) (430) | 108.0 (97.0, 124.0) (300) | 107.5 (92.0, 126.0) (130) | 0.48 |
| DABP (mmHg) | 73.0 (63.0, 84.0) (430) | 73.0 (63.0, 84.5) (300) | 73.0 (62.0, 82.0) (130) | 0.44 |
| MAP (mmHg) | 85.0 (76.0, 96.0) (430) | 85.0 (76.0, 97.0) (300) | 85.0 (75.0, 94.0) (130) | 0.36 |
| Serum Creatinine (mg/dL) | 1.4 (1.0, 1.9) (432) | 1.3 (1.0, 1.8) (300) | 1.6 (1.1, 2.2) (132) | 0.003 |
| eGFR (mL/min/1.73m^2) | 54.0 (37.0, 65.0) (376) | 57.5 (41.0, 73.0) (258) | 49.1 (28.6, 60.0) (118) | <0.001 |
| Hemoglobin (g/dL) | 12.6 (10.9, 14.4) (429) | 12.7 (11.2, 14.6) (297) | 12.6 (10.2, 14.0) (132) | 0.065 |
| Lactate (mmol/L) | 2.1 (1.4, 4.4) (216) | 1.9 (1.3, 3.5) (132) | 2.3 (1.6, 5.4) (84) | 0.024 |
| Platelets (10^3/uL) | 210.0 (161.0, 263.0) (429) | 216.0 (169.0, 264.0) (299) | 189.0 (152.0, 262.0) (130) | 0.043 |
| WBC (10^3/uL) | 9.2 (6.9, 13.9) (429) | 9.1 (6.9, 12.5) (299) | 10.5 (7.0, 16.2) (130) | 0.01 |
| **Context of Impella 5.5 Support** |  |  |  |  |
| Received MCS prior to first Impella implant | 34.6% (147/425) | 26.4% (77/292) | 52.6% (70/133) | <0.001 |
| ECMO + Other | 39.5% (58/147) | 27.3% (21/77) | 52.9% (37/70) |  |
| Other | 60.5% (89/147) | 72.7% (56/77) | 47.1% (33/70) |  |
| Cardiogenic shock prior to Impella device implant | 80.3% (355/442) | 76.7% (234/305) | 88.3% (121/137) | 0.005 |
| Cardiogenic Shock Setting |  |  |  | <0.001 |
| AMICS | 25.0% (89/356) | 18.9% (44/233) | 36.6% (45/123) | — |
| PCCS | 6.7% (24/356) | 3.4% (8/233) | 13.0% (16/123) | — |
| HFCS | 39.0% (139/356) | 45.5% (106/233) | 26.8% (33/123) | — |
| Myocarditis | 0.8% (3/356) | 0.9% (2/233) | 0.8% (1/123) | — |
| Other | 12.9% (46/356) | 13.3% (31/233) | 12.2% (15/123) | — |
| Unknown | 15.4% (55/356) | 18.0% (42/233) | 10.6% (13/123) | — |
| **Duration** |  |  |  |  |
| Time from index admission to Impella 5.5 (days) | 5 (2, 11) (442) | 6 (2, 11) (305) | 5 (2, 10) (137) | 0.37 |
| Duration of Impella 5.5 Support (days) | 9 (5, 17) (442) | 10 (6, 20) (305) | 8 (4, 14) (137) | <0.001 |
| Total Cohort Support Duration (days) | 5,871 | 4,405 | 1,466 | — |
| Length of Hospital Stay (days) | 29 (18, 47) (442) | 35 (23, 52) (305) | 17 (9, 25) (137) | <0.001 |

*p-values represent comparison between ≤ 14 days vs. > 14 days with either t-test, Wilcoxon rank sum test, fishers exact test, or Chi-square, as appropriate.

**Supplemental Table 5. Disposition at Discharge**

| Characteristics | All  N = 443 | DOS ≤ 14 Days  N = 306 | DOS > 14 Days  N = 137 | P-Value* | |
| --- | --- | --- | --- | --- | --- |
| **Survival at discharge, % (n/N)** |  |  |  |  |  |
| **Outcome at Discharge** |  |  |  | <0.001 | |
| Expired | 31.0% (137/442) | 34.8% (106/305) | 22.6% (31/137) |  | |
| Heart Transplant | 17.0% (75/442) | 8.9% (27/305) | 35.0% (48/137) |  | |
| Left Ventricular Assisted Device | 9.5% (42/442) | 8.9% (27/305) | 10.9% (15/137) |  | |
| Native Heart Survival | 42.5% (188/442) | 47.5% (145/305) | 31.4% (43/137) |  | |

*p-values represent comparison between ≤ 14 days vs. > 14 days with either t-test, Wilcoxon rank sum test, fishers exact test, or Chi-square, as appropriate. DOS, Duration Of Support

**Supplemental Figure 2.** **Survival stratified by heart replacement therapies: Kaplan-Meier survival estimates through 1-year.**

**Supplemental Figure 3.** **Freedom from All-Cause Mortality stratified by heart replacement therapies: Conditional Survival at Discharge through 1-year**

**Supplemental Figure 4.** **Survival from time of Impella 5.5 implant stratified by Treatment Strategy: Kaplan-Meier survival estimates through 1-year.**

**
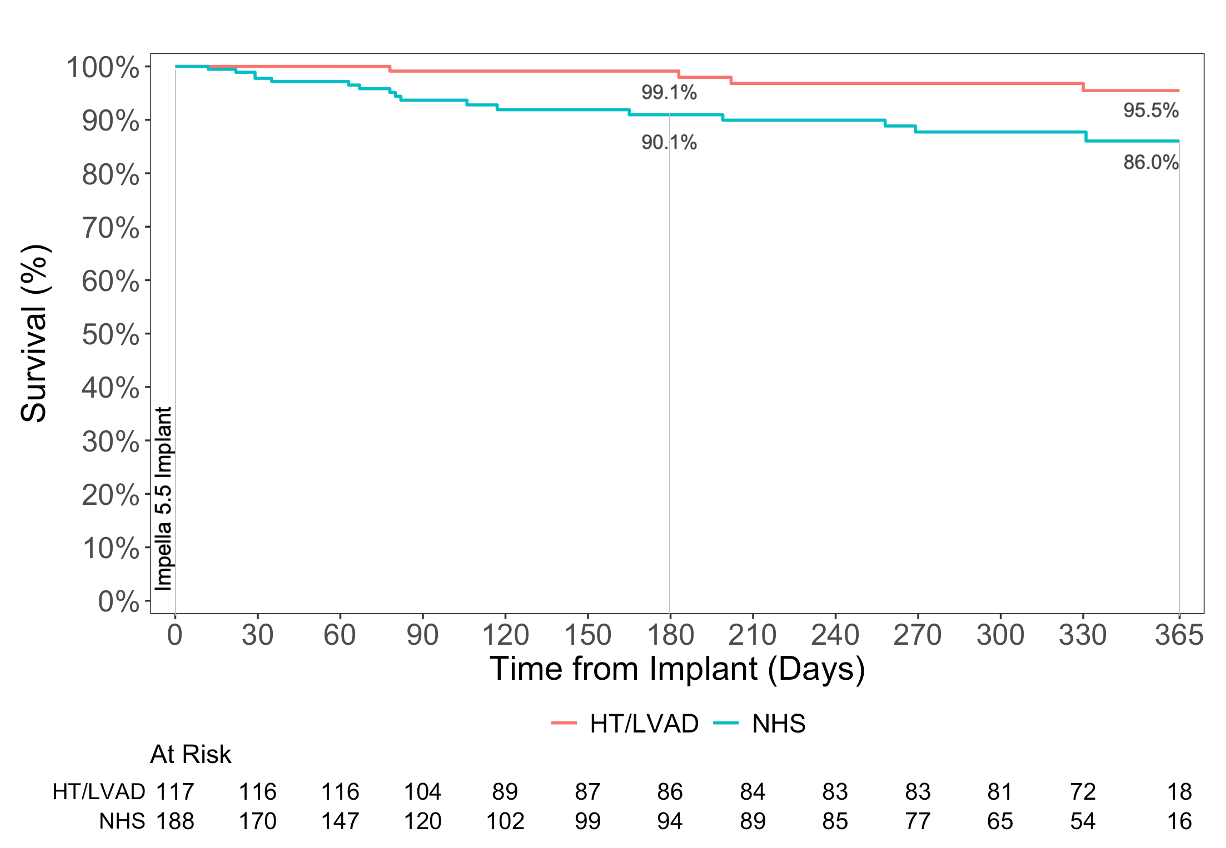
**

Treatment strategy: Heart replacement therapy use (HT, LVAD); Native Heart Survival(NHS)

**Supplemental Figure 5. Freedom from All-Cause Mortality stratified by Treatment Strategy: Conditional Survival at Discharge through 1-year**

**
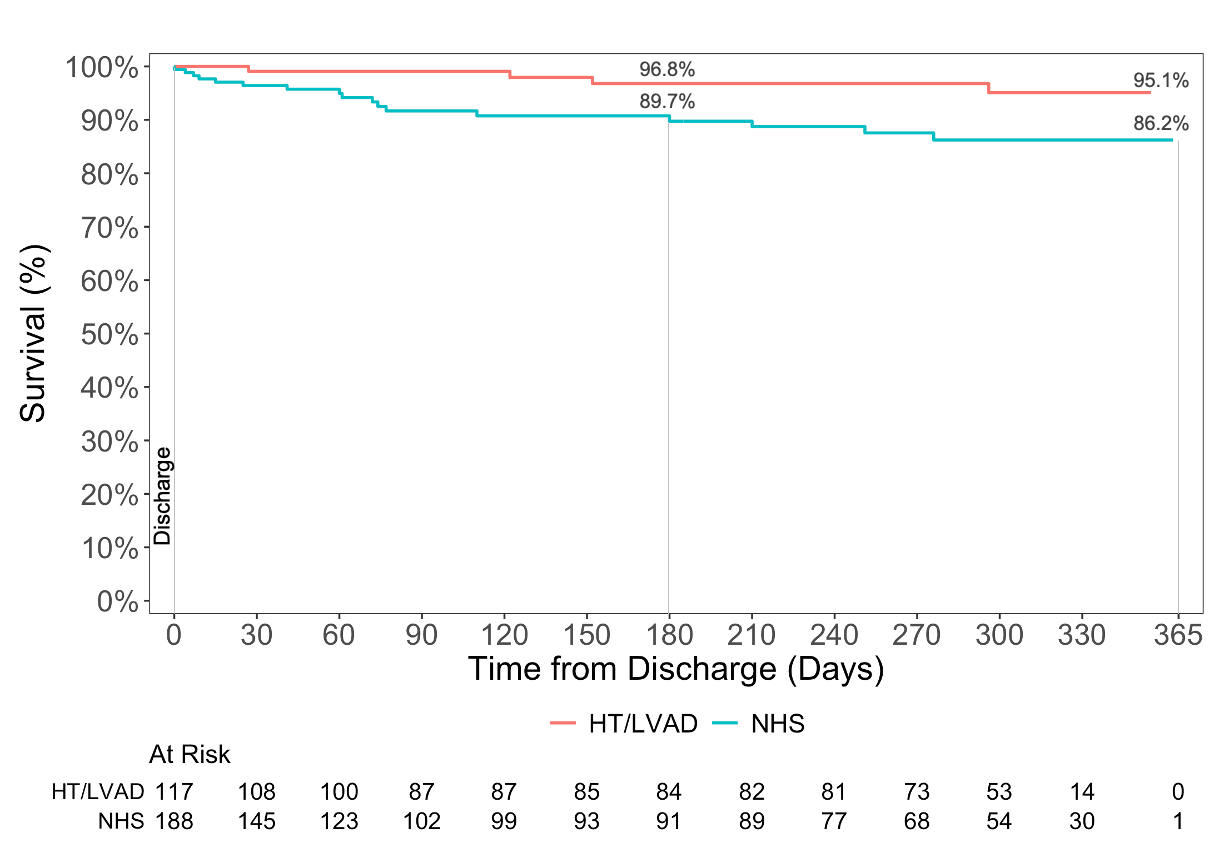
**

Treatment strategy: Heart replacement therapy use (HT, LVAD); Native Heart Survival(NHS)

**Supplemental Figure 6. EAER for Device-related Serious Adverse Events Subtypes**

Device-related AEs identified by site as those possibly, probably, or definitely related to the Impella 5.5 device. Adverse event rates were compared using a Poisson regression model using the number of events as dependent variable and log(subject total duration of support). P-values represent comparison between DOS ≤ 14 Days and > 14 Days.

**Supplemental Figure 7. Matched Analysis Covariate Balance**

Patients matched based on age, sex, race, prior MCS, and an exact match on etiology. Prior to balancing the absolute standardized difference can be seen exceeding the threshold of 0.10. After matching, all covariates are within the 0.10 indicative of a matched patient population between DOS ≤ 14 Days and DOS > 14 Days.


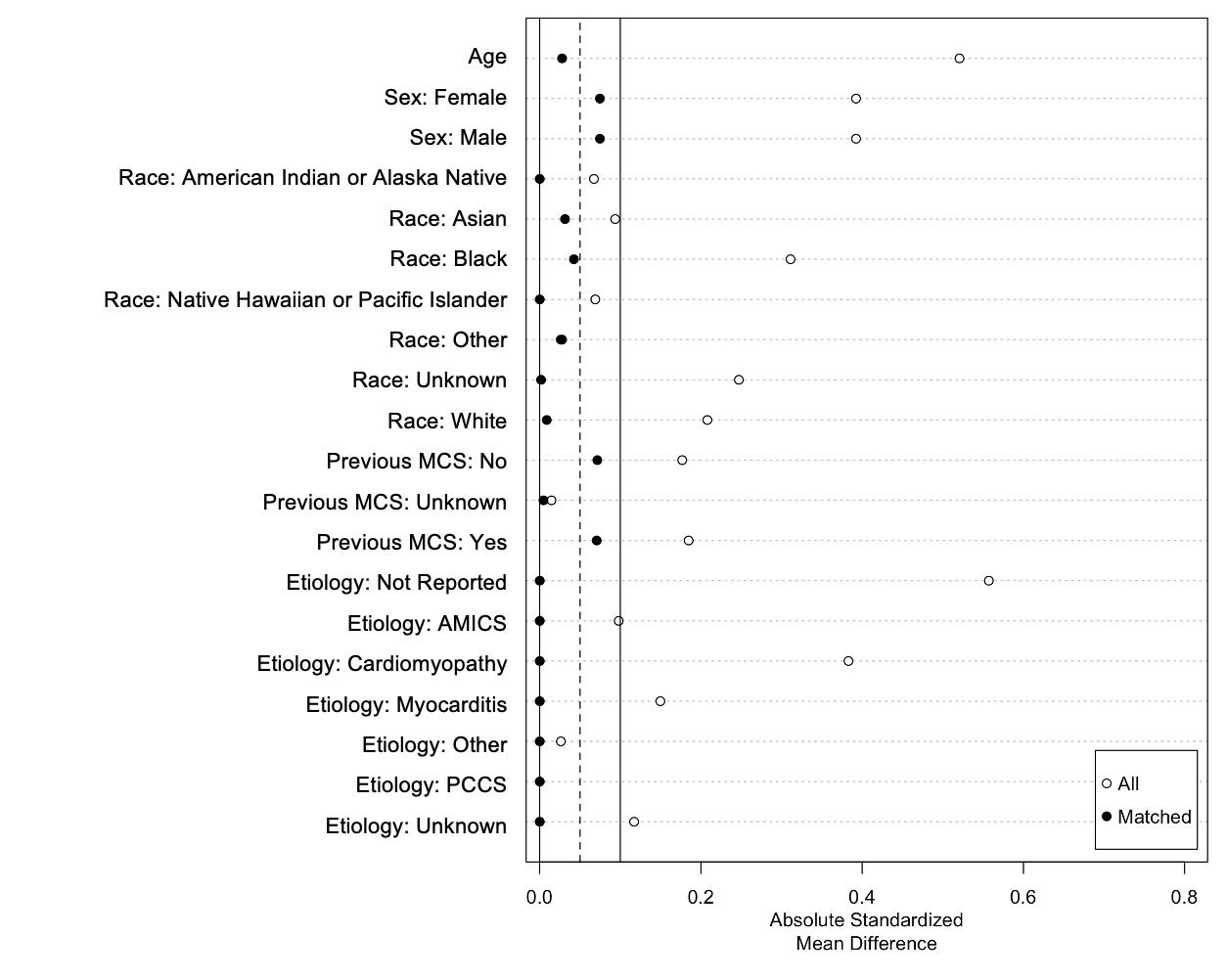


**Supplemental Figure 8. Freedom from All-Cause Mortality: Conditional Survival at Discharge through 1-year in Matched Cohort**

Kaplan-Meier survival estimates among patients that survived through 1-year in matched cohort. P-values represent comparison between DOS ≤ 14 Days and > 14 Days.


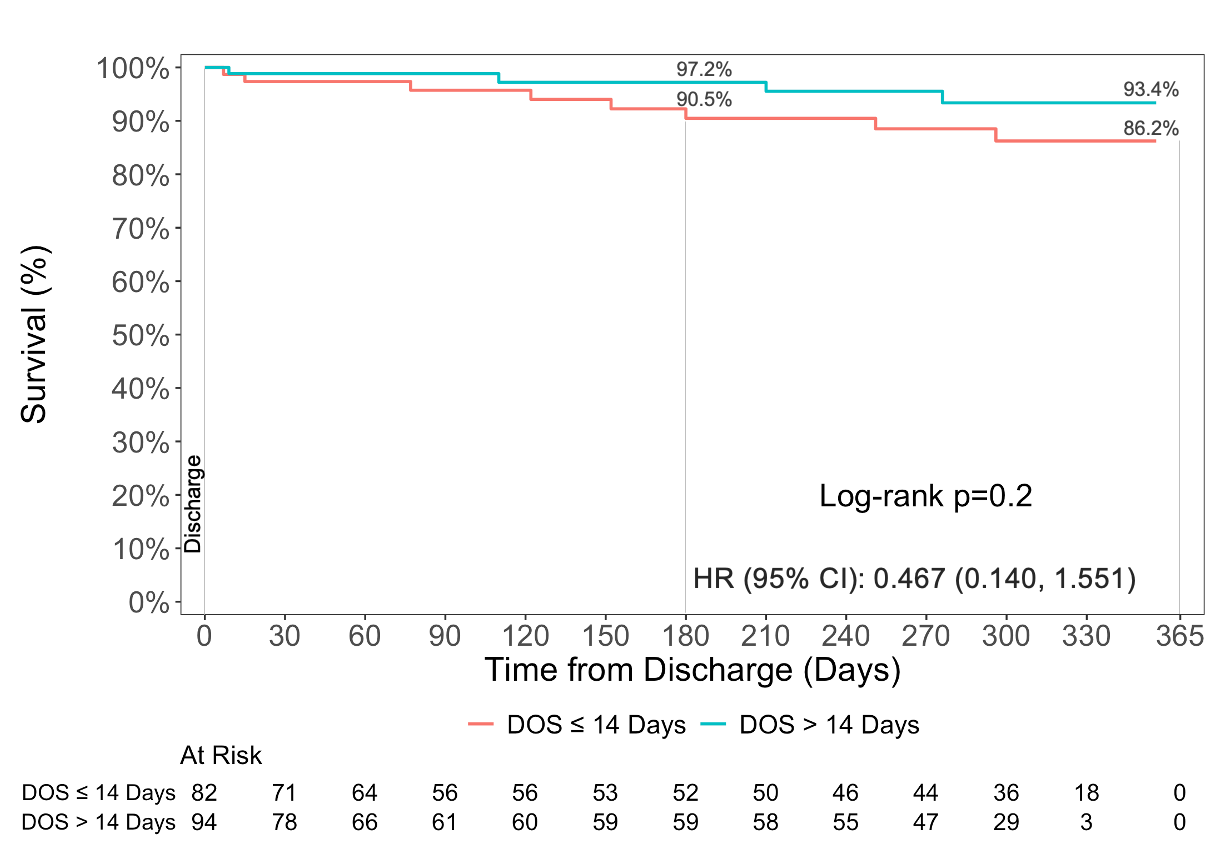


**Supplemental Table 6. Disposition at Discharge in Matched Cohort**

| Characteristics | All  N = 245 | DOS ≤ 14 Days  N = 123 | DOS > 14 Days  N = 122 | P-Value* |
| --- | --- | --- | --- | --- |
| **Outcome at Discharge** |  |  |  | 0.002 |
| Expired | 28.2% (69/245) | 33.3% (41/123) | 23.0% (28/122) |  |
| Heart Transplant | 24.1% (59/245) | 13.8% (17/123) | 34.4% (42/122) |  |
| LVAD | 13.1% (32/245) | 15.4% (19/123) | 10.7% (13/122) |  |
| Native Heart Survival | 34.7% (85/245) | 37.4% (46/123) | 32.0% (39/122) |  |

*p-values represent comparison between ≤ 14 days vs. > 14 days with either t-test, Wilcoxon rank sum test, fishers exact test, or Chi-square, as appropriate.

**Supplemental Figure 9. EAER for Serious Adverse Events Subtypes in Matched Cohort**

Adverse event rates were compared using a Poisson regression model using the number of events as dependent variable and log(subject total duration of support). P-values represent comparison between DOS ≤ 14 Days and > 14 Days.
